# Genomic evidence for the widespread presence of GH45 cellulases among soil invertebrates

**DOI:** 10.1111/mec.17351

**Published:** 2024-05-07

**Authors:** Hannah Muelbaier, Freya Arthen, Gemma Collins, Thomas Hickler, Karin Hohberg, Ricarda Lehmitz, Yannick Pauchet, Markus Pfenninger, Anton Potapov, Juliane Romahn, Ina Schaefer, Stefan Scheu, Clément Schneider, Ingo Ebersberger, Miklós Bálint

**Affiliations:** ^1^ Applied Bioinformatics Group, Inst. of Cell Biology and Neuroscience Goethe University Frankfurt am Main Germany; ^2^ LOEWE Centre for Translational Biodiversity Genomics Frankfurt am Main Germany; ^3^ Manaaki Whenua – Landcare Research Auckland New Zealand; ^4^ Senckenberg Biodiversity and Climate Research Centre Frankfurt am Main Germany; ^5^ Department of Physical Geography Goethe University Frankfurt/Main Germany; ^6^ Senckenberg Museum of Natural History Görlitz Görlitz Germany; ^7^ Insect Symbiosis, Max Planck Institute for Chemical Ecology Jena Germany; ^8^ Institute for Molecular and Organismic Evolution Johannes Gutenberg University Mainz Germany; ^9^ Senckenberg Museum for Natural History Görlitz Görlitz Germany; ^10^ German Centre for Integrative Biodiversity Research (iDiv) Halle‐Jena‐Leipzig Leipzig Germany; ^11^ International Institute Zittau TUD Dresden University of Technology Zittau Germany; ^12^ Institute of Insect Biotechnology Justus‐Liebig University Giessen Germany; ^13^ Animal Ecology University of Goettingen Goettingen Germany; ^14^ J.F. Blumenbach Institute of Zoology and Anthropology University of Goettingen Goettingen Germany

**Keywords:** arthropods, comparative genomics, decomposition, global change, horizontal gene transfer

## Abstract

Lignocellulose is a major component of vascular plant biomass. Its decomposition is crucial for the terrestrial carbon cycle. Microorganisms are considered primary decomposers, but evidence increases that some invertebrates may also decompose lignocellulose. We investigated the taxonomic distribution and evolutionary origins of GH45 hydrolases, important enzymes for the decomposition of cellulose and hemicellulose, in a collection of soil invertebrate genomes. We found that these genes are common in springtails and oribatid mites. Phylogenetic analysis revealed that cellulase genes were acquired early in the evolutionary history of these groups. Domain architectures and predicted 3D enzyme structures indicate that these cellulases are functional. Patterns of presence and absence of these genes across different lineages prompt further investigation into their evolutionary and ecological benefits. The ubiquity of cellulase genes suggests that soil invertebrates may play a role in lignocellulose decomposition, independently or in synergy with microorganisms. Understanding the ecological and evolutionary implications might be crucial for understanding soil food webs and the carbon cycle.

## 1 INTRODUCTION

Most photosynthetically bound carbon on land ends up in woody plants as lignocellulose, a composite of cellulose, hemicelluloses, lignin and pectin. The decomposition of lignocellulose occurs predominantly in soils, which returns most of this carbon into the atmosphere (Post et al., 1990). Terrestrial ecosystems currently sequester about 29% of the anthropogenic carbon emissions, which implies an important but not fully understood role of terrestrial carbon cycling for climate regulation (Cragg et al., 2015). Microorganisms, especially bacteria and fungi, encode glycoside hydrolase cocktails for lignocellulose degradation in their genomes (Cragg et al., 2015), and are considered the main actors of decomposition (Bradford et al., 2017; Crowther et al., 2019; Pausas & Bond, 2020). The contribution of animals to decomposition of lignocellulose—beyond purely mechanical shredding—remains less understood. Experiments have shown that the presence of soil invertebrates can increase litter mass loss by up to 50% (García‐Palacios et al., 2013). It is estimated that they decompose more deadwood in tropical forests than free‐living microorganisms (Griffiths et al., 2019). Nevertheless, the mechanisms behind decomposition performed by soil invertebrates remain obscure and the ability of soil animals to degrade composite polysaccharides without relying on gut symbionts remains a long‐standing debate in soil ecology (Berg et al., 2004; Cragg et al., 2015).

It was originally assumed that lignocellulose degradation by animals was entirely ‘outsourced’ to the gut microbiome (Briones, 2018; García‐Palacios et al., 2013). However, evidence is emerging that at least some invertebrates, such as molluscs, crustaceans and phytophagous insects, can synthesize cellulase enzymes themselves (Busch et al., 2019; Chang & Lai, 2018; Cragg et al., 2015; Griffiths et al., 2021; Han et al., 2022; Kern et al., 2013; King et al., 2010; Shelomi et al., 2014; Watanabe et al., 1998). Scattered evidence also exists for the expression of active endogenous cellulases by distantly related soil invertebrates, for example, the earthworm *Pheretima hilgendorfi* (Nozaki et al., 2009), the Antarctic springtail *Cryptopygus antarcticus* (Hong et al., 2014), as well as few oribatid mites and other springtails (Busch et al., 2019). Based on these individual findings, we hypothesize that a larger fraction of soil invertebrates than previously thought may be directly contributing to decomposition of lignocellulose in dead plant matter in soils using endogenous cellulases, with or without relying on a microbiome. Given their global abundance and diversity in many soil ecosystems (FAO et al., 2020; Phillips et al., 2019; Potapov et al., 2023; van den Hoogen et al., 2019), soil invertebrates could, therefore, have an important but so far overlooked role in the terrestrial carbon cycle which is distinct from the decomposition ability of microorganisms. To evaluate whether endogenous decomposition ability is a common feature shared by the main groups of soil invertebrates, we screened a diverse set of newly sequenced genomes of Collembola, Enchytraeidae, Gamasina, Myriapoda, Nematoda, Oribatida and Tardigrada for the presence and evolutionary origins of cellulase genes. We further investigated the hypothesis that cellulase genes in invertebrates were acquired by horizontal gene transfer (HGT) from microorganisms. Cellulases are classified into three different categories: Endoglucanases (EC 3.2.1.4) break down cellulose in smaller randomly sized fragments. Exoglucanases (EC 3.2.1.91) release cellobiose and cellotriose from the ends of cellulose molecules. Cellobiases (EC 3.2.1.21) convert cellobiose into glucose. Common to all cellulases is their activity to hydrolyse glycoside bonds, and the CAZy database currently lists 187 different families of glycoside hydrolases (Drula et al., 2022). Here, we focus on GH45 cellulases because these were already described in hexapods, and they were found to be functional (*Cryptopygus antarcticus* and *Phaedon cochleariae* (Girard and Jouanin 1999; Song et al. 2008; Busch et al., 2019)).

## 2 MATERIALS AND METHODS

### 2.1 Domain architectures of invertebrate GH45‐type cellulases

Reviewed evidence exists for the presence of GH45‐type cellulases in the Antarctic springtail (*Cryptopygus antarcticus*; Collembola, UniprotID D3GDK4) and the mustard beetle (*Phaedon cochleariae*; Insecta, UniprotID O97401). Since these experimentally confirmed cellulases harbour a Glyco‐hydro 45 Pfam domain (PF02015) (Bankevich et al., 2012), we restricted our analysis to cellulase orthologues that carry this Pfam domain. Pfam domains were annotated with hmmscan from the HMMER package (Finn et al., 2015) using Pfam version 32 and applying the default e‐value cut‐off of 0.01.

### 2.2 Genome assembly pipeline

The genome assemblies provided by the MetaInvert Project (Bioproject ID: PRJNA758215) cover a phylogenetically diverse set of soil‐living invertebrates collected from the field or obtained from cultures. Short read Illumina sequencing (300 bp paired‐end) with the NovaSeq 6000 platform was done at Novogene Europe (Cambridge, UK), reads were trimmed with Trimmomatic, human contaminating reads were filtered with Kraken2 (Wood et al., 2019) and contig assembly was done with SPAdes (Bankevich et al., 2012). The resulting contigs were taxonomically assigned with Blobtools2 (Challis et al., 2020) using the NCBI non‐redundant protein database as a reference, and only contigs with an assignment to the phylum of the target species together with unassigned contigs were kept. Redundancy reduction and scaffolding were done with Redundans (Pryszcz & Gabaldón, 2016), and genome assembly completeness was assessed with BUSCO (v 4.1.4) using the precomputed metazoan (obd10) reference set. The genome data and metadata were published and further analysed by Collins et al. (2023) (in press: 10.1038/s42003‐023‐05621‐4). Collection (geographic coordinates) and vouchering data of the used specimens are provided by Collins et al. (2023). For our orthologue search, we selected genomes with at least 50% BUSCO completeness (176 assemblies, Table S2).

### 2.3 RefSeq gene set collection

We downloaded gene sets for all 18,412 taxa represented in the NCBI RefSeq Genome release 207 (O'Leary et al., 2016). The resulting taxon collections comprised 16,401 bacteria, 910 archaea, 409 fungi, 262 invertebrates and 430 vertebrates. The taxon list together with the accession numbers is provided in Table S1.

### 2.4 Taxonomic assignment and contaminant detection

To rule out that fungal or bacterial contaminations of the underlying genome assemblies are responsible for the animal cellulase orthologues (Steinegger & Salzberg, 2020), we followed a multi‐step procedure. First, during the invertebrate genome assembly (described in Collins et al., 2023, not part of this paper), all contigs were used as input for a Blobtools2 (Challis et al., 2020) analysis using the NCBI non‐redundant protein and nucleotide databases as a reference. Only contigs assigned to the phylum of the target species and unassigned contigs were passed on to the downstream analyses. Second, we taxonomically classified each of the detected invertebrate orthologues. In brief, we used the orthologue sequence as a query for a Diamond version 2.0.13 (Buchfink et al., 2015) search against the NCBI non‐redundant protein database (downloaded January 2022). From the resulting hit list, we excluded the trivial hit against itself and then assigned the query sequence to the last common ancestor of the taxa within a 10% bit score margin of the best hit (Huson et al., 2016). A sequence was flagged as a putative contaminant if its taxonomic assignment was not placed on the lineage from the species whose genome was analysed to the root of the tree of cellular life. The workflow is implemented into the software package taXaminer (https://github.com/BIONF/taxaminer), and it provided no evidence for a foreign origin of these sequences (Table S3). As a showcase example, we further investigated the origin of two GH45 cellulase orthologues. In the mite *Carabodes femoralis*, the orthologue is located on a short contig with no neighbouring genes. We taxonomically assigned this gene to the Acariformes. In the springtail *Pogonognathellus longicornis*, the GH45 cellulase is located together with other genes on a contig of 41 kbp in length. The cellulase and neighbouring genes on the same contig were consistently assigned to Entomobryomorpha as a result (Figure S5).

### 2.5 Orthology‐based phylogenetic profiles of fungal GH45 cellulase

Profile‐based targeted orthologue searches in annotated gene sets were performed with fDOG (Birikmen et al., 2021) using the GH45 cellulase of the fungus *Rhizoctonia solani* (NCBI Accession XP_043186467.1) as the seed. For the training of the initial profile Hidden Markov model, we used the parameter *‐‐minDist genus* and ‐‐*maxDist phylum* limiting the number of training sequences to 6 (see Table S4 for more information). Candidate orthologues were filtered for the presence of the Pfam glyco‐hydro 45 domain (PF02015; see Table S5 for a list of discarded orthologues). Orthologue search in the unannotated MetaInvert genome assemblies was performed with the fDOG extension fDOG‐Assembly. In brief, genomic regions likely containing a GH45‐type cellulase were identified with a tBLASTn search using the consensus sequence included in the initial core gh45 core group from fDOG as query. The hit region was extended by 500 nucleotides on either side and genes in the resulting candidate genomic region were annotated with MetaEuk v5.34c21f2 (Levy Karin et al., 2020) using the OMA database (released in December 2021; Nguyen et al., 2015) as the reference database for the gene prediction. The corresponding protein sequences were then tested for orthology using the routines of fDOG and afterwards, features were annotated with FAS (Dosch et al., 2023). The fDOG‐assembly workflow is available from https://github.com/BIONF/fDOG/tree/fdog_goes_assembly. We completed the taxon collection by adding four individual genomes publicly available on GenBank (Table S6) and performed the same orthologue searches described above. The results from fDOG and fDOG‐Assembly were merged and visualized with PhyloProfile (Tran et al., 2018).

### 2.6 GH45 cellulase gene tree reconstruction

To investigate the evolutionary history of the GH45 cellulases, we used the identified orthologues for a gene tree reconstruction. If the orthologue search obtained more than one co‐orthologue, we used the one that is most similar to the seed protein from *R. solani* for the tree reconstruction. Sequences were aligned with Muscle v3.8.1551 (Edgar, 2004) and alignment columns comprising more than 50% gaps were removed with a custom perl script. The resulting multiple sequence alignment was used as input for a maximum likelihood tree reconstruction with IQ‐TREE (Nguyen et al., 2015) version 1.6.8. Branch support was assessed with 1000 bootstrap replicates using the ultrafast bootstrap approach. The SH‐aLRT branch test was performed, and the optimal number of cores was automatically detected via IQ‐TREE (− nt AUTO). All gene trees were visualized with iTOL (Letunic & Bork, 2021). Animals are paraphyletic in this tree, and a topology test using the AU test (Shimodaira & Hasegawa, 1999) confirmed that a tree with monophyletic animal cellulases explained the data significantly worse (p‐AU = 9.2E−4). A second gene tree containing all identified orthologues and co‐orthologues was reconstructed with the same approach. The two tree files in Newick format are available as Supplement Files F1 and F2.

### 2.7 Phylogenies of springtails and oribatids

Phylogenies of springtail and oribatid organisms included in the MetaInvert project were computed separately with a supermatrix approach. BUSCO version 5.4.2 (Simão et al., 2015) with the precomputed BUSCO Arthropoda gene set (db10) was used to search for orthologues in all genome assemblies. BUSCO genes were discarded from the analysis which were present in less than 75% of all species. Multiple sequence alignments were computed with MAFFT using local pairwise alignment and at maximum 1000 iterations (7.481) (Katoh & Standley, 2013), trimmed with clipkit (1.3.0) (Steenwyk et al., 2020) and concatenated with FASconCAT‐G (1.04) (Kück & Longo, 2014). Four phylogenetic trees per taxon group were reconstructed with IQ‐TREE (Nguyen et al., 2015). Branch support was assessed with 1000 bootstrap replicates using the ultrafast bootstrap approach. The best‐fit model was Q.insect + F + R9 for oribatids, and Q.insect + F + R10 for springtails, automatically chosen by ModelFinder according to BIC. The final consensus tree was computed with splitstree (4.19.0) (Huson & Bryant, 2006) by summarizing the four IQ‐TREEs into a consensus tree. The consensus trees were outgroup‐rooted using *Sarcoptes scabiei* (GCA_020844145.1) for oribatids, and *Machilis hrabei* (GCA_003456935.1), *Drosophila albomicans* (GCA_009650485.1) and *Tyrophagus putrescentiae* for springtails as outgroups. The species trees and reproductive mode of the corresponding species (taken from Collins et al. 2023) were visualized with iTOL. Chi‐squared tests were performed with R.

### 2.8 Correspondence of GH45 tree and phylogenies

The BUSCO‐based phylogenies of springtails and oribatids were outgroup‐rooted and merged for the comparison with the GH45 cellulase gene tree. The tanglegram matches taxa by links and was computed with R using the packages phytools v1.4‐0 (Revell, 2012) and castor v1.7.6 (Louca & Doebeli, 2018).

### 2.9 3D structure comparison

3D structures of *R. solani* GH45 cellulase and of the endoglucanase V from *Humicola insolens* were retrieved from precomputed predictions from UniProt (accession number A0A0B7FQX1 and P43316). The 3D structure of the *F. candida GH45 cellulase* was locally computed with AlphaFold (Jumper et al., 2021). The structures were visualized and compared with VMD (Humphrey et al., 1996) and the extensions MultiSeq (Roberts et al., 2006) in combination with the alignment tool STAMP (Russell & Barton, 1992). The active site was identified by highlighting in yellow the catalytic residues found by Davies et al., 1993. A structure‐guided sequence alignment of all three GH45 sequences was computed with expresso from the t‐coffee package (Armougom et al., 2006) (Figure S4).

## 3 RESULTS

### 3.1 Endogenous GH45 cellulases are mainly found in fungi and invertebrates

**FIGURE 1:**
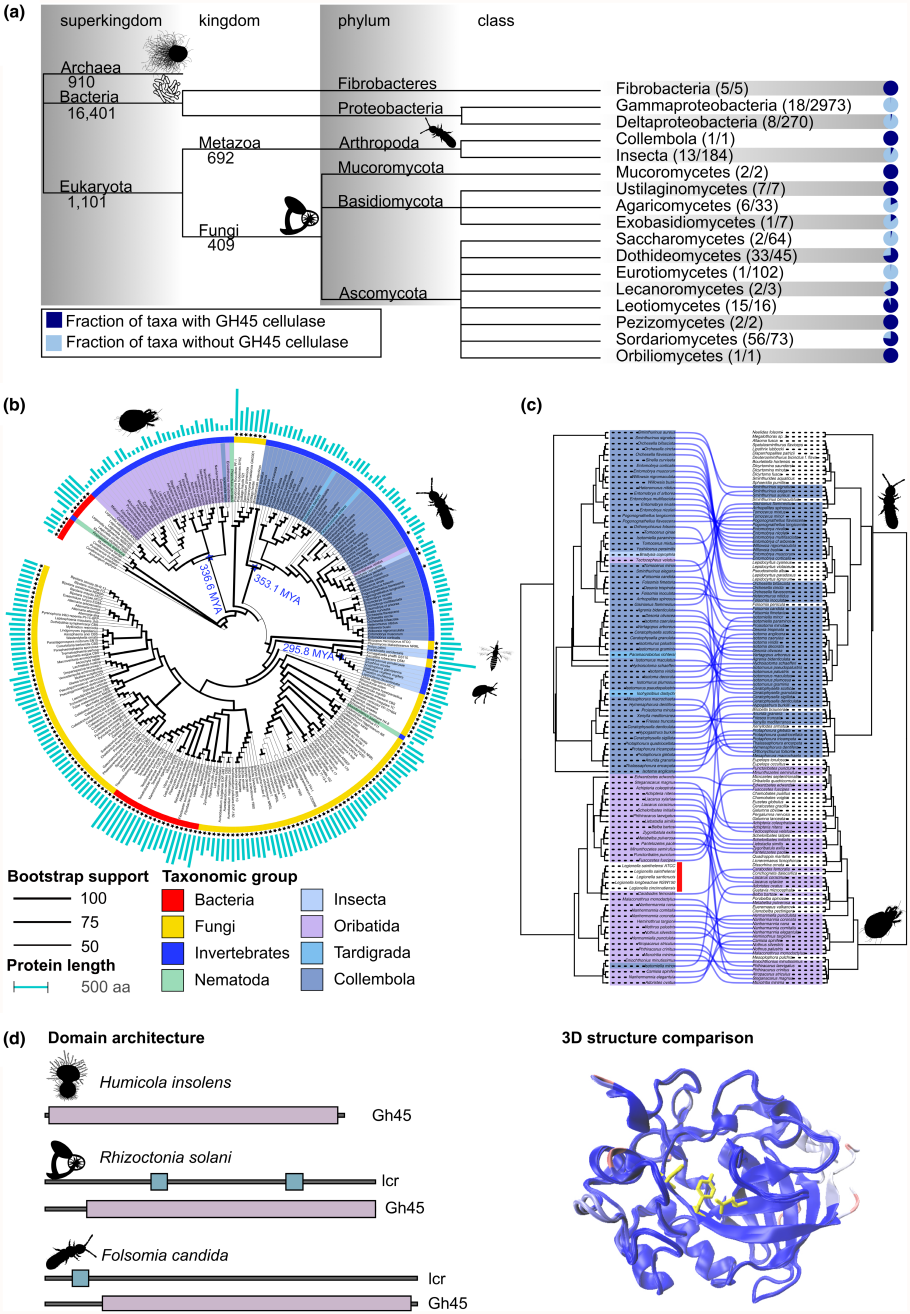
GH45 cellulases on the tree of life. (a) Abundance of GH45 cellulases across the three domains of life; (b) Maximum likelihood phylogeny of the GH45 cellulase family. Branch lengths are not drawn to scale and line weights indicate percent bootstrap support. Species represented by a genome assembly in the NCBI RefSeq or GenBank databases are indicated by an asterisk. Pictograms identify the four main soil invertebrate clades: oribatid mites, springtails, thrips and beetles (clockwise). Bars indicate the protein length of the respective GH45 cellulase in amino acids. Internal node labels provide age estimates of the respective clades (Kumar et al., 2022); (c) Tanglegram between the GH45 gene tree (left) and a phylogenomic reconstruction of the species tree (right). Only cellulases identified in oribatids and springtails are considered. Lines connect the GH45 cellulases in the gene tree with the corresponding taxa they were identified in the species tree; (d) Left: Comparison of protein domain architecture of two fungal (*H. insolens* (Uniprot ID: P43316), *R. solani* (Uniprot ID: A0A0B7FQX1)) and one invertebrate GH45 cellulase (*F. candida* (NCBI Acc: XP_021945337.1)). lcr—low complexity region; GH45—Pfam Glyco‐hydro 45 domain (PF02015). Right: 3D structure alignment of the same three GH45 cellulases. Thirty amino acids from the N termini of the *R. solani* and *F. candida* proteins are not considered in the structural comparison because the corresponding alpha helix could not be confidently placed in the structure by AlphaFold (per‐residue confidence score (pLDDT) <70; (Tunyasuvunakool et al., 2021)). Structural similarity is colour coded and ranges from red (very low) to very high (blue). The catalytic residues located in the active groove of the *H. insolens* cellulase (Davies et al., 1993) are highlighted in yellow. A structure‐guided amino acid sequence alignment is shown in Figure S4 and reveals the conservation of these catalytic residuals in the other two proteins.

We used a fungal sequence as a starting point to obtain a comprehensive overview of the taxonomic distribution of the GH45 cellulase family. In an initial screen, we considered all species that are represented by a genome assembly in NCBI RefSeq. We decided to base this analysis on fungal GH45 cellulase because (i) existing results suggest that invertebrate GH45 cellulases were likely horizontally transferred from fungi (Busch et al., 2019) and (ii) fungal GH45 cellulases have been thoroughly functionally characterized (Berto et al., 2019; Bharadwaj et al., 2020). We searched for orthologues to fungal GH45 cellulases in 16,401 bacteria, 910 archaea and 1101 eukaryotes (Table S1). The resulting phylogenetic profile revealed that GH45 cellulases are abundant only in fungi, where 128 out of 409 investigated taxa possess at least one GH45 cellulase gene. However, they are limited to individual taxonomic groups (Figure 1a). In contrast, only 31 out of 16,401 bacterial species were found to harbour a GH45 cellulase. Next to fungi, orthologues to GH45 cellulases were largely confined to animals (14 out of 692) (Figure 1a). We found no evidence that these comprise fungal contaminations (see taxonomic assignment and contaminant detection in Section 4). In metazoans, the majority of orthologues were found in arthropods, and they were completely absent in vertebrates.

### 3.2 Most springtails and oribatid mites possess endogenous GH45 cellulases

The analysis of publicly available genomes sheds light on the general distribution of GH45 cellulases across the Tree of Life. However, it lacks the resolution to investigate the occurrence of this cellulase in soil invertebrates. We extended the analysis by including novel genome assemblies for an additional 176 species representing a diverse selection of soil invertebrates (NCBI BioProject PRJNA758215) into the analysis. This revealed a high occurrence of GH45 cellulases in springtails (56/78 analysed species) and in oribatid mites (33/54 analysed species, Table S2). Additionally, we detected cellulases in Coleoptera and Thysanoptera (2/2 species, Table S2). In three out of nine nematode species we also found GH45 cellulases, whereas none were found in 30 representatives of Chilopoda and Diplopoda (Table S2).

GH45 cellulases are present in three of four known main springtail lineages (Poduromorpha, Entomobryomorpha and Symphypleona), missing only from the earliest branching Neelipleona. Within Symphypleona and Entomobryomorpha, cellulases are consistently absent in one clade each (Figure S1). GH45 cellulases are present in almost all representatives of the basal clades in Oribatida (Enarthronota, Mixonomata, Holosomata; Table S2). In contrast, they were missing in half of the investigated species from the later‐branching Brachypylina (Figure S2). Integrating the presence–absence pattern of GH45 cellulases with the reproduction mode of the respective species (sexually vs. parthenogenetic) reveals no significant correlation for Oribatida and Collembola respectively (χ^2^ test, *p* > .05). Besides Oribatida, we investigated a second mite taxon, the Gamasina, which are represented by two species in our data sets (Table S2). We found in neither species a GH45 cellulase.

### 3.3 The evolutionary history of invertebrate GH45 cellulases is complex

We reconstructed the phylogenetic relationships of the sequences identified both in RefSeq assemblies and in soil invertebrate genomes (Figure 1b) to better understand the evolutionary trajectory resulting in the present‐day distribution of soil invertebrate GH45 cellulases. In the resulting tree, 104 out of 107 animal proteins are placed in only four distinct and taxonomically largely homogenous clades, one each for the Collembola, Oribatida, Thysanoptera and Coleoptera. On a larger scale, we found that the invertebrate cellulase clades are embedded into an evolutionary background formed by mostly fungal and very few bacterial sequences. This leaves metazoans as a taxonomic group paraphyletic with respect to their cellulases and indicates that GH45 cellulases have been independently introduced into the animal kingdom at least four times, very likely from fungal donors. We noted that several bacterial cellulases, all from the genus *Legionella*, form a monophyletic clade placed within the diversity of the oribatid cellulases. This finding suggests a horizontal transfer of an animal gene into a bacterial clade. However, the corresponding branch is very long (1.78 substitutions per site; SupplData) and further investigations will be necessary to consolidate this hypothesis. To assess whether the evolutionary relationships of the invertebrate cellulases agree with those of the species they were found in, we compared the phylogeny from oribatid mites and springtails with the reconstructed cellulase gene tree (Figure 1c). The two trees display in large parts agreeing branching patterns, which is expected for intrinsic cellulases. However, they clearly differ in other parts. To trace down the reason for these differences, we reconstructed the invertebrate cellulase tree this time considering all detected GH45 co‐orthologues. This revealed a highly dynamic and complex evolutionary history of the invertebrate GH45 cellulase family involving numerous lineage‐specific gene duplications and losses (Figure S3).

### 3.4 Fungal and invertebrate GH45 cellulases are structurally conserved

In a last analysis, we investigated whether the detected invertebrate GH45 cellulases are likely functional. We compared the domain architectures and the predicted 3D protein structures between GH45 cellulases of the fungus *Rhizoctonia solani* and the springtail *Folsomia candida* to that of the fungus *Humicola insolens*, whose GH45 cellulase has been functionally and structurally characterized before (Davies et al., 1993). All three enzymes agree in their domain architecture and have highly similar 3D structures (Figure 1d). Moreover, we found the three catalytic residues in the substrate‐binding active grove initially described by Davies et al. (1993) to be conserved across the proteins (Figure S4). Together, this strongly indicates that all three proteins can hydrolyse glycoside bonds.

## 4 DISCUSSION

Most cellulases discovered to date in metazoan genomes belong to the GH45 family (Busch et al., 2019), endo‐β‐1,4‐glucanases which hydrolyse cellulose, lichenin and cereal β‐D‐glucans (EC 3.2.1.4). Individual members of this family appear specific for degrading xyloglucan (EC 3.2.1.151). However, Busch et al. (2019) have shown that the tested GH45 cellulases show no substrate specificity and break down both cellulose and xyloglucan. It was hypothesized that invertebrate GH45 cellulases were repeatedly obtained via HGT from fungal donors (Busch et al., 2019). Our search in 18,412 RefSeq genomes revealed that only 31 bacterial species harbour a GH45‐type cellulase. This is in line with the earlier hypothesis about the evolutionary roots of this enzyme within fungi (Busch et al., 2019; Palomares‐Rius et al., 2014). The taxonomic distribution of GH45 cellulases was non‐uniform in metazoans, with most orthologues being found in arthropods. GH45 cellulases were reported in marine bivalves (Okmane et al., 2022) which feed on phytoplankton and on particulate matter. We could not evaluate these findings, as genomes of the respective species are not yet included in the NCBI RefSeq database. Our results confirm previous research which did not find evidence for GH45 presence in vertebrates (Chang & Lai, 2018). The screening of new soil invertebrate genomes uncovered novel GH45 cellulase presence patterns. GH45 genes were found in well over half of the investigated springtail and oribatid mite species.

Springtails form a basal hexapod group abundant across the globe, especially in cold regions (Potapov et al., 2023). They are known as fungal feeders, but also consume detritus and fresh plant materials (Potapov et al., 2020). While the orthologue search showed the presence of GH45 cellulase in springtail lineages Poduromorpha, Entomobryomorpha and Symphypleona, it is missing from the earliest branching Neelipleona. Neelipleona feed on detritus colonized by fungi and bacteria rather than on plant remains (Potapov et al., 2022), and thus may not require the activity of an endogenous cellulase. A single acquisition event post‐dating the divergence of the Neelipleona may explain this observation. However, since Neelipleona are represented only by a few taxa, the conclusion on cellulase absence should be taken cautiously. Within Symphypleona and Entomobryomorpha, cellulases are consistently absent in one clade each. The absence of cellulase genes is unexpected in the lucerne flea *Sminthurus viridis* that feeds on live leaf tissue (Greenslade & Ireson, 1986), and in the families of Dicyrtomidae, Bourletiellidae and Sminthuridae which consume mainly fresh plant materials (Potapov et al., 2020, 2022). The lack of cellulase genes suggests that these taxa either outsource cellulose decomposition to their midgut microbiome, rely on other GH families or do not digest cellulose. Besides these exceptions, the presence of GH45 cellulase genes seems to be a common trait among springtails.

Mites are the most numerous arthropods on land (Rosenberg et al., 2023), with most representatives in soil ecosystems belonging to Oribatida. Oribatid mites can have diverse feeding strategies, but they mostly feed on leaf litter at different decomposition stages and on microorganisms (Maraun et al., 2023). Our results showed that GH45 cellulases are present all over the basal clades of Oribatida while they were missing in half of the Branchypylina, which diversified later in the course of oribatid evolution. One interesting finding is that GH45 cellulases are missing especially in sexually reproducing mites, while they are present in other sexually reproducing taxa. However, the correlation between the reproduction mode and the presence of the GH45 cellulases is not statistically significant. An alternative hypothesis is that parthenogenetic oribatid mites tend to occupy lower trophic positions and typically function as primary decomposers, opposed to secondary decomposers feeding predominantly on microorganisms (Fischer et al., 2014). However, ecological interpretation of these patterns is difficult since we do not know if species without GH45 cellulase genes contain other classes of cellulases, digest cellulose with the help of their microbiome or are indeed incapable of cellulose digestion. In general, the complex pattern of GH45 presence is similar to the low phylogenetic conservatism of ecological traits in oribatids, such as feeding mode (Potapov et al., 2022).

As expected from previous findings (Kirsch et al., 2014), we detected cellulases in Coleoptera. GH45 cellulases were completely absent in the genomes of Chilopoda and Diplopoda. The latter was surprising as Diplopoda are a key litter‐feeding soil invertebrate group (Joly et al., 2020; Potapov et al., 2022). However, it was previously shown that the millipede *Telodeinopus aoutii* relies on its gut microbiome for lignocellulose degradation (Sardar et al., 2022). GH45 cellulases were also absent in Gamasina mites which are predators and therefore might not benefit from cellulose degradation. The first report of endogenous cellulases in Thysanoptera suggests that our analysis uncovers only the tip of the iceberg. We expect that taxonomically broad genome sequencing of eukaryotes promoted, for example, by the Earth BioGenome Project (Formenti et al., 2022; Lewin et al., 2022) will recover further animal groups in possession of enzymes targeting lignocellulose decomposition.

Taken together, our data suggest an early acquisition of a GH45 cellulase during the diversifications of both springtails and oribatids, instead of repeated horizontal transfer events. This implies that the possession of a GH45 cellulase is an ancestral trait in these groups. Similar to our results, cellulase acquisition was shown to be important for the diversification of herbivorous beetles (Kirsch et al., 2014). Differences in the GH45 cellulase gene tree from the oribatid and springtail species trees likely result from a highly dynamic evolution of the GH45 cellulase repertoire. Lineage‐specific gene duplications and losses have partially disconnected the evolutionary history of the contemporary cellulase genes from the phylogeny of the species they are found in (Figure 1c; Figure S3). Lineage‐specific duplications have been described for other cellulases (Shelomi et al., 2016; Shin et al., 2022), and differential duplicate loss has been shown to result in gene tree–species tree incongruencies (Parey et al., 2020). The presence of cellulases detected in thrips suggests that processes similar to those in Oribatida and Collembola might have been important also during the evolution of other arthropod groups.

We want to emphasize that the widespread presence of cellulase genes in soil invertebrates does not exclude that many species also rely on cellulolytic enzymes from microorganisms. Lignocellulose is a highly complex composite of cellulose, hemicellulose, lignin and pectin and its decomposition requires complex enzymatic cocktails (Cragg et al., 2015). For example, enzymes from the microbiome and the host work synergistically for lignocellulose degradation in the pill bug *Armadillidium vulgare* (Bredon et al., 2018). Wood decomposition in termites is also a synergistic activity carried out together by insect and gut symbiont enzymes (Brune, 2014). In addition, transient microbiota, consumed together with lignocellulosic fragments, may also be secreting relevant enzymes. Decomposition performed by invertebrates in synergy with gut microorganisms would be a very flexible system capable of fast adaptation to environmental changes.

Cellulase presence in invertebrate genomes is not a proof of function. However, several lines of evidence point towards functionality. First, domain architecture of GH45 cellulases in fungi (*H. insolens* and *R. solani*) and the springtail *F. candida* are similar, and the fungal enzymes also have similar predicted 3D structures with the cellulases found by us (Figure 1d). Second, the catalytic residues that are important for the enzymatic function (Davies et al., 1993) are conserved in the two fungi and the springtail. Finally, orthologues with conserved domain architectures were retained over hundreds of millions of years of evolution in springtails and oribatids. This suggests that little change has occurred in the trophic niche and position of springtails and oribatid mites in soil food webs since their evolutionary origins. This view is further supported by the presence of both taxonomic groups in the first fossil soils (Schaefer & Caruso, 2019; Shear et al., 1984). Taken together, these are strong indications that GH45 cellulases in springtails and oribatids perform cellulose decomposition. Future work to experimentally evaluate the functional properties of soil invertebrate cellulases (Song et al., 2017) should consider all glycoside hydrolase genes, as gene duplication events may have led to substrate diversification (Busch et al., 2019; Shin et al., 2022), with duplicates being able to break down other polysaccharides like xyloglucan, mannans or xylan. While orthologues can be identified bioinformatically, functional properties need to be confirmed experimentally by expressing these enzymes heterologously, and test their substrate specificity on cellulose and hemicellulose polysaccharides.

Strong evidence exists that fungal GH45 cellulase genes have been repeatedly horizontally transferred to invertebrates. Several properties can explain why the initially foreign genes were integrated as an evolutionarily stable part of the recipient species' metabolic network. First, cellulases, as secreted, gut‐acting enzymes (Fischer et al., 2013) do not depend on existing physiological pathways and their regulation for proper functioning in the recipient organism. Second, such enzymes likely reach their correct extracellular destination directly after the successful transfer of the cellulase gene, its incorporation into the genome and its translation, because the signals of protein export generally work independent of origin in most other taxa, even over vast evolutionary distances (Clérico et al., 2008). Third, the reaction catalysed by cellulases yields products that can serve as a beneficial fitness‐relevant resource in any organism, because the necessary downstream pathways are ubiquitously present. Supporting these arguments, genes transferred horizontally are often secreted proteins (Savory et al., 2015; Undheim & Jenner, 2021).

The horizontal acquisition of cellulases and of other plant cell wall‐degrading enzymes likely was a key event driving the evolutionary emergence of herbivory in arthropods (Wybouw et al., 2016). It coincided, for example, with the massive radiation of Phytophaga, the most species‐rich clade of beetles (Busch et al., 2019), and in adaptation to lignocellulose‐rich diets in crustaceans (King et al., 2010). The long‐term evolutionary preservation of GH45 genes suggests that cellulases likely confer fitness benefits also to soil invertebrates. These benefits may come from direct use of plant carbohydrate resources, although some theories imply that soil invertebrates are limited rather by access to proteins, but not by access to carbohydrates. The ability to degrade complex polysaccharides may also provide access to more nutritious, protein‐rich cytosols or microorganisms colonizing the inside of plant cells, such as saprotrophic fungi, which are considered as major dietary components of both collembolans and oribatids (Pollierer & Scheu, 2021). We expect that cellulase presence/absence patterns across large taxon collections integrated with phenotypic traits of the respective species and the trophic niches they inhabit (Maraun et al., 2023; Potapov et al., 2022) will provide insights into the functional ecology and evolution of soil invertebrates.

It was previously shown that individual invertebrate species are capable of degrading lignocellulose independent from their gut microbiome (King et al., 2010). The widespread presence of GH45 cellulases in springtails and oribatid mites suggests that such endogenous cellulolytic abilities are substantially more common in invertebrates than it is generally appreciated. In addition to bacteria and fungi, invertebrates should, therefore, be considered a third evolutionarily and ecologically distinct group with such capability. This has important consequences for our understanding of soil food webs and the soil carbon cycle. Fungi compared to bacteria are known to react differently to environmental change such as experimental warming (Melillo et al., 2017) or habitat degradation (Zhou et al., 2018). This results from key differences in life‐history strategies, for example, growth rates or nutrient use (Jansson & Hofmockel, 2020). Their differential reaction to environmental change influences decomposition as distinct taxa determine the rate and biochemical pathways of organic matter processing (Crowther et al., 2019). For example, fungal‐based food webs in soils, and the processes of C and N loss they govern, are more resistant against and are better adaptable to drought than bacterial food webs (de Vries et al., 2012). Fungi accordingly contribute more to litter decomposition than bacteria under drought conditions (Ullah et al., 2023). Soil invertebrates react differently to environmental change compared to microorganisms (Sünnemann et al., 2021). Given key differences in life‐history strategies among soil invertebrates, bacteria and fungi, a comparison of endogenous cellulolytic capabilities of soil invertebrates and microorganisms is an important direction for future research. We hypothesize that global change has a more detrimental impact on decomposition performed by soil invertebrates, given their lower effective population sizes and adaptive elasticity (Lanfear et al., 2014; Pauls et al., 2013). It might be essential to consider all three systematic groups and their differences for a better integration of below‐ground processes into ecosystem models (Chertov et al., 2017; Deckmyn et al., 2020; Filser et al., 2016) including global carbon models (Friedlingstein et al., 2022), and for better predictions of soil carbon and nutrient cycling.

## AUTHOR CONTRIBUTIONS

MB, IE, HM, SS, IS, AP, MP, YP and GC conceptualized the study. IE, HM, FA, MB, CS, JR, RL, KH and GC contributed methodology and materials. HM, FA and IE performed the analyses and visualized the results. IE supervised the analyses. MB and IE wrote the original draft of the manuscript. HM, GC, TH, KH, RL, YP, MP, AP, JR, IS, SS, CS, IE and MB reviewed and finalized the manuscript. All data needed to evaluate the conclusions in the paper are present in the paper and/or the Supplementary Materials. Genome data will be available via NCBI BioProject PRJNA758215.

## CONFLICT OF INTEREST STATEMENT

The authors declare that they have no competing interests.

## BENEFIT‐SHARING STATEMENT

The authors have no benefits to report.

## Supporting information


Data S1

Table S1

Files F1‐F2

## Data Availability

Genome data: Only publicly available genome data were used for this research. The gene sets originated from NCBI RefSeq or GenBank (see Table S1), while the genome assemblies of soil‐living invertebrates were taken from Bioproject PRJNA758215. Metadata: Metadata associated with this study are available via figshare (https://doi.org/10.6084/m9.figshare.c.7121512.v1).
