# Supplementary material for: Genomic evidence for the widespread presence of GH45 cellulases among soil invertebrates
Source: Mol Ecol. 2024 May 7;33(20):e17351. doi: 10.1111/mec.17351 (PMC13084967; doi:10.1111/mec.17351)
Supplement: Supplementary file 1 — Data S1 [file MEC-33-e17351-s001.docx]

Supplementary Materials

Supplementary Figures


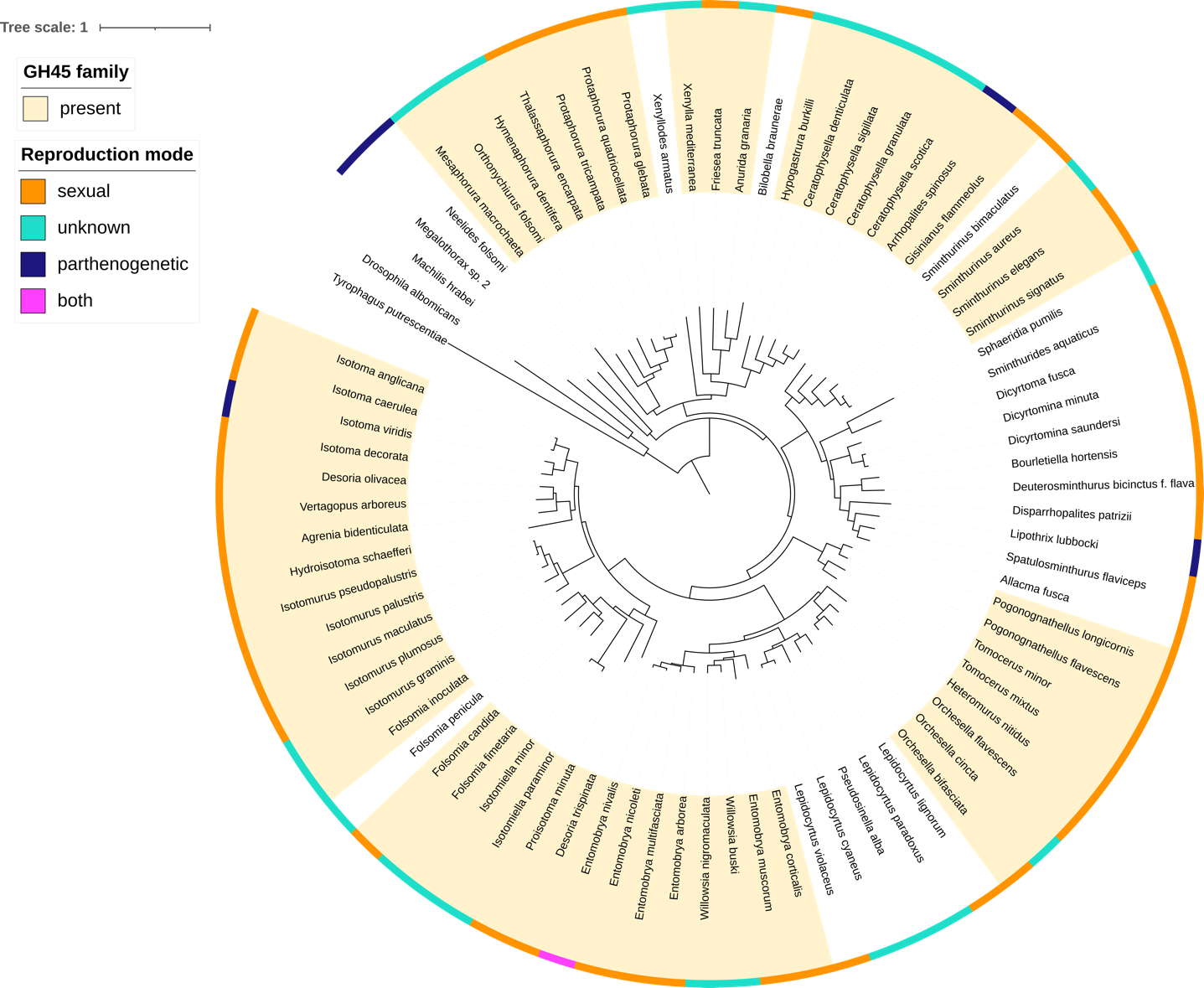


Fig. S1. Maximum likelihood phylogeny of springtails. Species with GH45 cellulase are highlighted in yellow. The outer ring indicates the reproductive mode of the respective species.


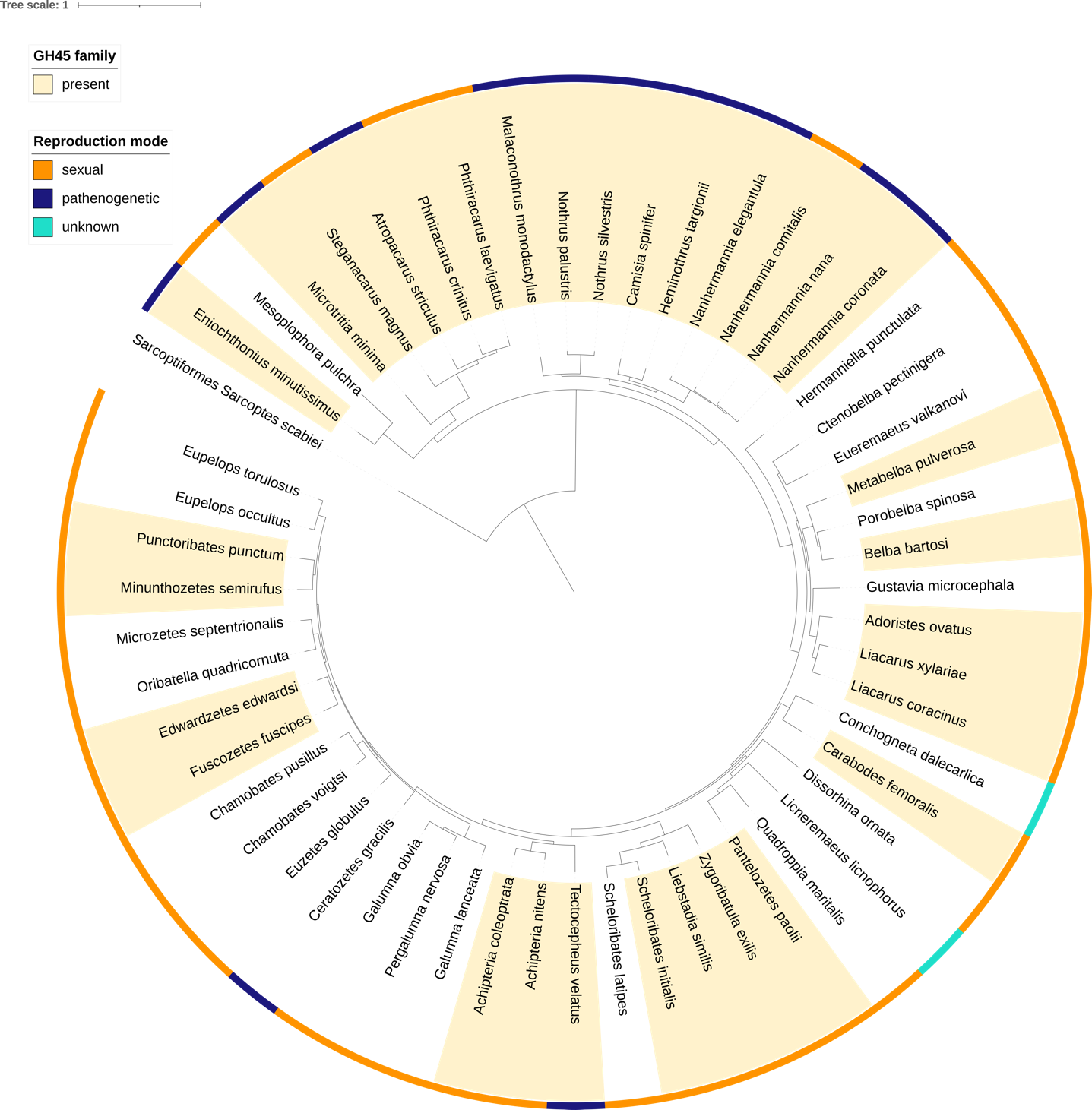


**Fig. S2.** Maximum likelihood phylogeny of oribatid mites. Species with GH45 cellulase are highlighted in yellow. The outer ring indicates the reproductive mode of the respective species.

**Fig. S3**. Phylogenetic relationships of the invertebrate GH45 cellulases. The cladogram represents the unrooted maximum likelihood relationships of all GH45 cellulases identified in this study. Arcs connect proteins residing in the genome of the same species. These proteins have been identified as co-orthologs of the *Rhizoctonia solani* GH45 cellulase and emerged from a gene duplication event that occurred after a primordial animal GH45 cellulase was acquired, likely from a fungal donor. The distance of the connected GH45 cellulases in the tree approximates the evolutionary age of the respective gene duplication events.


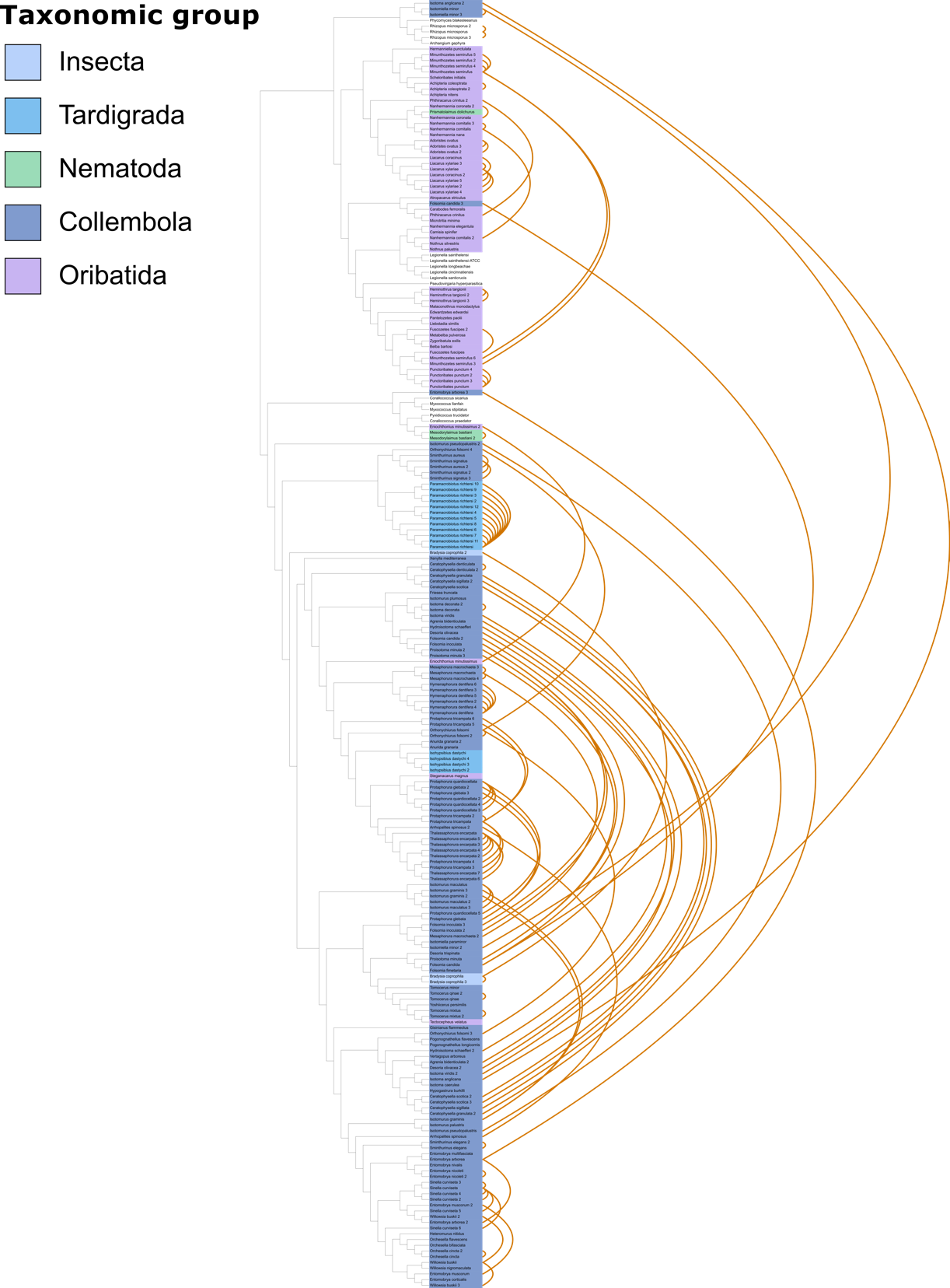


**Fig. S3**. Phylogenetic relationships of the invertebrate GH45 cellulases. The cladogram represents the unrooted maximum likelihood relationships of all GH45 cellulases identified in this study. Arcs connect proteins residing in the genome of the same species. These proteins have been identified as co-orthologs of the *Rhizoctonia solani* GH45 cellulase and emerged from a gene duplication event that occurred after a primordial animal GH45 cellulase was acquired, likely from a fungal donor. The distance of the connected GH45 cellulases in the tree approximates the evolutionary age of the respective gene duplication events.


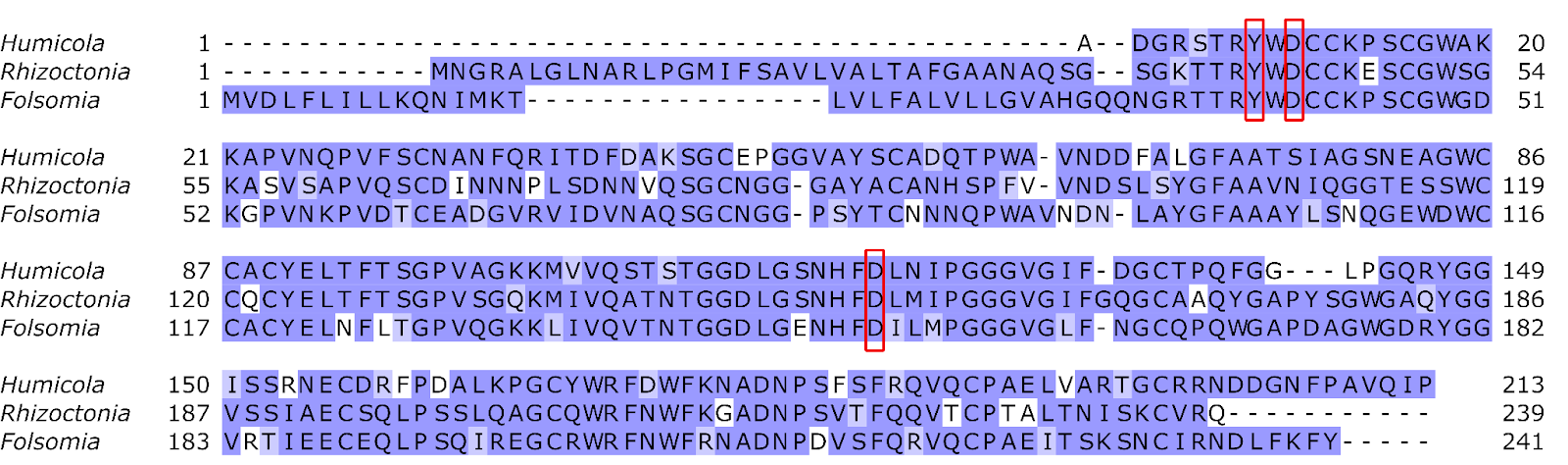


**Fig S4.** 3D structure guided sequence alignment of GH45 cellulases. The multiple sequence alignment reveals that the three catalytic amino acids Y(8), D(10) and D(121) are conserved in the two fungal and the invertebrate GH45 cellulases. *Humicola* - *Humicola insolens*; *Rhizoctonia* - *Rhizoctonia solani*; *Folsomia* - *Folsomia candida*


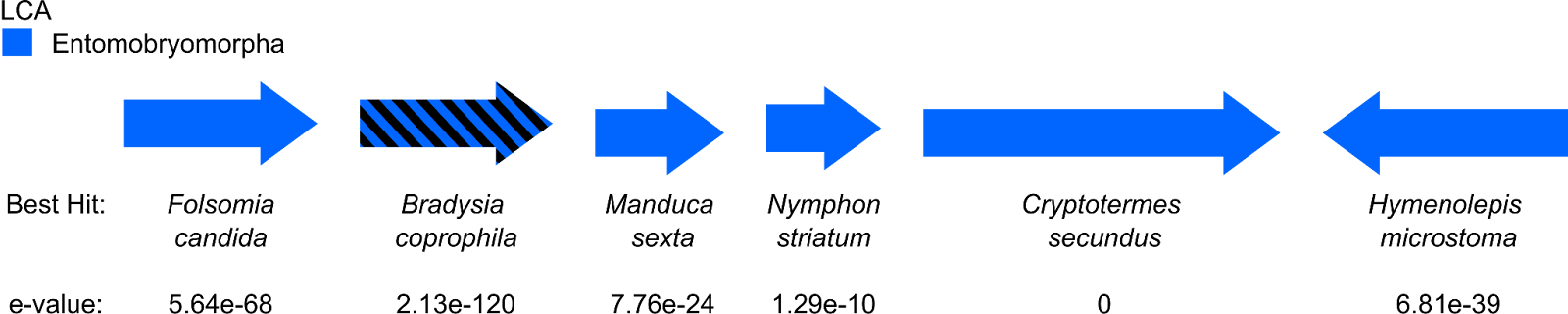


**Fig. S5.** Taxonomic assignment of the genes surrounding the GH45 cellulase in *Pogonognathellus longicorns*. The hatched arrow represents the GH45 cellulase, blue arrows represent the flanking genes with the arrow tip indicating the direction of transcription. The protein sequence encoded by each gene was used as a query for a Diamond search against the NCBI non-redundant protein database. The best hit taxon together with the corresponding e-value are given below each gene. All genes are taxonomically assigned to the Entomobryomorpha.

Supplementary Tables

**Table S1. Screened RefSeq genomes**. Bacterial, archaeal, fungal, invertebrate and vertebrate genomes screened for the presence of GH45 cellulase orthologs. (uploaded separately)

**Table S2. Screened MetaInvert genomes**. Soil invertebrate genomes screened with fDOG-Assembly for the presence of GH45 cellulase orthologs.

| **libid** | **countgroup** | **Scientific name** | **taxid** | **busco completness** | **Gh45 cellulase** |
| --- | --- | --- | --- | --- | --- |
| a_17 | Chilopoda | *Cryptops parisi* | 173049 | 0,77 | Absent |
| a_84 | Chilopoda | *Geophilus carpophagus* | 173285 | 0,82 | Absent |
| P3_3 | Chilopoda | *Geophilus flavus* | 856749 | 0,72 | Absent |
| a96 | Chilopoda | *Geophilus truncorum* | 173284 | 0,84 | Absent |
| a_4 | Chilopoda | *Haplophilus subterraneus* | 173289 | 0,65 | Absent |
| a_5 | Chilopoda | *Henia vesuviana* | 126936 | 0,8 | Absent |
| a_21 | Chilopoda | *Pachymerium ferrugineum* | 115410 | 0,84 | Absent |
| a_83 | Chilopoda | *Stenotaenia linearis* | 1569481 | 0,76 | Absent |
| a42 | Chilopoda | *Strigamia acuminata* | 1255758 | 0,76 | Absent |
| a39 | Chilopoda | *Strigamia crassipes* | 1428135 | 0,79 | Absent |
| a_23 | Chilopoda | *Strigamia transsilvanica* | 1579475 | 0,7 | Absent |
| a_66 | Collembola | *Agrenia bidenticulata* | 1933610 | 0,82 | Present |
| MI_479 | Collembola | *Allacma fusca* | 39272 | 0,84 | Absent |
| a_35 | Collembola | *Anurida granaria* | 187597 | 0,79 | Present |
| a107 | Collembola | *Arrhopalites spinosus* | 187608 | 0,6 | Present |
| a_42 | Collembola | *Bilobella braunerae* | 106916 | 0,57 | Absent |
| a102 | Collembola | *Bourletiella hortensis* | 574228 | 0,76 | Absent |
| P1_24 | Collembola | *Ceratophysella denticulata* | 928250 | 0,75 | Present |
| a_34 | Collembola | *Ceratophysella granulata* | 1218962 | 0,78 | Present |
| a52 | Collembola | *Ceratophysella scotica* | 187617 | 0,62 | Present |
| a49 | Collembola | *Ceratophysella sigillata* | 1218965 | 0,66 | Present |
| a72 | Collembola | *Desoria olivacea* | 370026 | 0,55 | Present |
| MI_424 | Collembola | *Desoria trispinata* | 1184801 | 0,61 | Present |
| a3_16 | Collembola | *Deuterosminthurus bicinctus f. flava* | 2041938 | 0,67 | Absent |
| a_31 | Collembola | *Dicyrtoma fusca* | 1385863 | 0,8 | Absent |
| a_32 | Collembola | *Dicyrtomina minuta* | 1387116 | 0,77 | Absent |
| a_51 | Collembola | *Dicyrtomina saundersi* | 438492 | 0,75 | Absent |
| P4_21 | Collembola | *Disparrhopalites patrizii* | 999999006 | 0,83 | Absent |
| a_64 | Collembola | *Entomobrya cf arborea* | 30001 | 0,77 | Present |
| a64 | Collembola | *Entomobrya corticalis* | 1503966 | 0,77 | Present |
| a_33 | Collembola | *Entomobrya muscorum* | 2041940 | 0,53 | Present |
| a_48 | Collembola | *Entomobrya nicoleti* | 2041941 | 0,7 | Present |
| a71 | Collembola | *Entomobrya nivalis* | 1387109 | 0,66 | Present |
| a108 | Collembola | *Entomobrya Typ multifasciata* | 247613 | 0,85 | Present |
| a103 | Collembola | *Folsomia candida* | 158441 | 0,91 | Present |
| a3_12 | Collembola | *Folsomia fimetaria* | 1387114 | 0,72 | Present |
| a_38 | Collembola | *Folsomia inoculata* | 2041942 | 0,77 | Present |
| MI_48 | Collembola | *Folsomia penicula* | 266765 | 0,59 | Absent |
| a60 | Collembola | *Friesea truncata* | 187628 | 0,7 | Present |
| a_40 | Collembola | *Gisinianus flammeolus* | 2449080 | 0,85 | Present |
| P2_15 | Collembola | *Heteromurus nitidus* | 254095 | 0,81 | Present |
| a98 | Collembola | *Hydroisotoma schaefferi* | 301519 | 0,85 | Present |
| a58 | Collembola | *Hymenaphorura dentifera* | 999999008 | 0,72 | Present |
| a_70 | Collembola | *Hypogastrura burkilli* | 1725397 | 0,71 | Present |
| a105 | Collembola | *Isotoma anglicana* | 247611 | 0,66 | Present |
| a_26 | Collembola | *Isotoma caerulea* | 308473 | 0,72 | Present |
| a_71 | Collembola | *Isotoma decorata* | 57735 | 0,57 | Present |
| P3_1 | Collembola | *Isotoma viridis* | 187635 | 0,75 | Present |
| P1_25 | Collembola | *Isotomiella minor* | 370032 | 0,83 | Present |
| a57 | Collembola | *Isotomiella paraminor* | 370031 | 0,58 | Present |
| a_36 | Collembola | *Isotomurus graminis* | 1184803 | 0,72 | Present |
| a_69 | Collembola | *Isotomurus maculatus* | 36143 | 0,66 | Present |
| a97 | Collembola | *Isotomurus palustris* | 36144 | 0,81 | Present |
| P1_26 | Collembola | *Isotomurus plumosus* | 1410395 | 0,53 | Present |
| a_25 | Collembola | *Isotomurus pseudopalustris* | 36142 | 0,79 | Present |
| a_53 | Collembola | *Lepidocyrtus cyaneus* | 247612 | 0,59 | Absent |
| MI_426 | Collembola | *Lepidocyrtus lignorum* | 707889 | 0,59 | Absent |
| a_27 | Collembola | *Lepidocyrtus paradoxus* | 49179 | 0,67 | Absent |
| P2_2 | Collembola | *Lepidocyrtus violaceus* | 707891 | 0,81 | Absent |
| a_46 | Collembola | *Lipothrix lubbocki* | 1387126 | 0,82 | Absent |
| a4_24 | Collembola | *Megalothorax sp. 2* | 2340290 | 0,83 | Absent |
| a68 | Collembola | *Mesaphorura macrochaeta* | 2651973 | 0,68 | Present |
| MI_445 | Collembola | *Neelides folsomi* | 332381 | 0,75 | Absent |
| a_29 | Collembola | *Orchesella bifasciata* | 576794 | 0,71 | Present |
| a53 | Collembola | *Orchesella cincta* | 48709 | 0,53 | Present |
| a_28 | Collembola | *Orchesella flavescens* | 48711 | 0,76 | Present |
| a106 | Collembola | *Orthonychiurus folsomi* | 2581074 | 0,79 | Present |
| a_49 | Collembola | *Pogonognathellus flavescens* | 511703 | 0,78 | Present |
| a54 | Collembola | *Pogonognathellus longicornis* | 707266 | 0,84 | Present |
| P2_17 | Collembola | *Proisotoma minuta* | 301521 | 0,84 | Present |
| a61 | Collembola | *Protaphorura glebata* | 187683 | 0,79 | Present |
| a_61 | Collembola | *Protaphorura quadriocellata* | 187683 | 0,72 | Present |
| P2_19 | Collembola | *Protaphorura tricampata* | 187683 | 0,74 | Present |
| a_41 | Collembola | *Pseudosinella alba* | 1302326 | 0,57 | Absent |
| a104 | Collembola | *Sminthurides aquaticus* | 281415 | 0,8 | Absent |
| a_39 | Collembola | *Sminthurinus aureus* | 1496267 | 0,84 | Present |
| P4_22 | Collembola | *Sminthurinus bimaculatus* | 187699 | 0,81 | Absent |
| P4_20 | Collembola | *Sminthurinus elegans* | 1190784 | 0,66 | Present |
| a100 | Collembola | *Sminthurinus signatus* | 2584529 | 0,86 | Present |
| a101 | Collembola | *Spatulosminthurus flaviceps* | 999999007 | 0,81 | Absent |
| a3_8 | Collembola | *Sphaeridia pumilis* | 212016 | 0,87 | Absent |
| P2_22 | Collembola | *Thalassaphorura encarpata* | 2583954 | 0,81 | Present |
| P2_23 | Collembola | *Tomocerus minor* | 187706 | 0,58 | Present |
| a55 | Collembola | *Tomocerus mixtus* | 58788 | 0,84 | Present |
| a_43 | Collembola | *Vertagopus arboreus* | 2041954 | 0,62 | Present |
| a_54 | Collembola | *Willowsia buski* | 1458441 | 0,77 | Present |
| a_55 | Collembola | *Willowsia nigromaculata* | 1302335 | 0,8 | Present |
| a99 | Collembola | *Xenylla mediterranea* | 2567731 | 0,78 | Present |
| a62 | Collembola | *Xenyllodes armatus* | 187716 | 0,71 | Absent |
| a94 | Diplopoda | *Chordeuma sylvestre* | 1569510 | 0,7 | Absent |
| a_14 | Diplopoda | *Cylindroiulus punctatus* | 61981 | 0,68 | Absent |
| a_96 | Diplopoda | *Glomeris hexasticha* | 1392624 | 0,66 | Absent |
| a_10 | Diplopoda | *Glomeris marginata* | 62006 | 0,67 | Absent |
| a_18 | Diplopoda | *Julus scandinavius* | 1008810 | 0,68 | Absent |
| a_93 | Diplopoda | *Julus scanicus* | 541046 | 0,72 | Absent |
| a_86 | Diplopoda | *Kryphioiulus occultus* | 1008825 | 0,72 | Absent |
| a91 | Diplopoda | *Megaphyllum sjaelandicum* | 52423 | 0,7 | Absent |
| a_95 | Diplopoda | *Melogona broelemanni* | 1147011 | 0,54 | Absent |
| a95 | Diplopoda | *Mycogona germanica* | 999999013 | 0,67 | Absent |
| a_13 | Diplopoda | *Ommatoiulus sabulosus* | 1008866 | 0,69 | Absent |
| a_92 | Diplopoda | *Ophyiulus pilosus* | 118470 | 0,61 | Absent |
| a_2 | Diplopoda | *Polydesmus angustus* | 1068628 | 0,72 | Absent |
| a_15 | Diplopoda | *Polydesmus complanatus* | 510027 | 0,84 | Absent |
| a_20 | Diplopoda | *Proteroiulus fuscus* | 88024 | 0,7 | Absent |
| a_91 | Diplopoda | *Rossiulus vilnensis* | 999999014 | 0,72 | Absent |
| a_12 | Diplopoda | *Xestoiulus laeticollis* | 1522044 | 0,74 | Absent |
| MI_473 | Enchytraeidae | *Cognettia cognettii* | 1502715 | 0,58 | Absent |
| a4_3 | Enchytraeidae | *Enchytraeus crypticus* | 913645 | 0,53 | Absent |
| MI_474 | Enchytraeidae | *Oconnorella tubifera* | 913705 | 0,6 | Absent |
| a35 | Gamasina | *Phytoseiulus persimilis* | 44414 | 0,8 | Absent |
| a36 | Gamasina | *Stratiolaelaps miles* | 406085 | 0,83 | Absent |
| a4_15 | Nematoda | *Acrobeloides thornei* | 96599 | 0,6 | Absent |
| P2_7 | Nematoda | *Aphelenchus avenae* | 70226 | 0,59 | Absent |
| MI_396 | Nematoda | *Discolaimus major* | 211252 | 0,61 | Absent |
| a85 | Nematoda | *Mesodorylaimus bastiani* | 344383 | 0,55 | Present |
| MI_395 | Nematoda | *Panagrellus redivivus* | 6233 | 0,55 | Absent |
| a3_9 | Nematoda | *Panagrolaimus detritophagus* | 310956 | 0,55 | Absent |
| P3_2 | Nematoda | *Phasmarhabditis papillosa* | 6243 | 0,64 | Absent |
| a75 | Nematoda | *Prionchulus punctatus* | 293874 | 0,58 | Present |
| D23 | Nematoda | *Prismatolaimus dolichurus* | 288633 | 0,53 | Present |
| P2_4 | Oribatida | *Achipteria coleoptrata* | 229769 | 0,78 | Present |
| P1_2 | Oribatida | *Achipteria nitens* | 229768 | 0,8 | Present |
| a1 | Oribatida | *Adoristes ovatus* | 708363 | 0,72 | Present |
| a2 | Oribatida | *Atropacarus striculus* | 229743 | 0,69 | Present |
| a3 | Oribatida | *Belba bartosi* | 2241992 | 0,8 | Present |
| a4 | Oribatida | *Camisia spinifer* | 198258 | 0,8 | Present |
| MI_457 | Oribatida | *Carabodes femoralis* | 229793 | 0,74 | Present |
| P1_32 | Oribatida | *Ceratozetes gracilis* | 1686620 | 0,77 | Absent |
| a6 | Oribatida | *Chamobates pusillus* | 503572 | 0,75 | Absent |
| a3_18 | Oribatida | *Chamobates voigtsi* | 198262 | 0,66 | Absent |
| P4_1 | Oribatida | *Conchogneta dalecarlica* | 999999009 | 0,81 | Absent |
| a7 | Oribatida | *Ctenobelba pectinigera* | 1401282 | 0,65 | Absent |
| P1_12 | Oribatida | *Dissorhina ornata* | 2202870 | 0,8 | Absent |
| a10 | Oribatida | *Edwardzetes edwardsi* | 2202872 | 0,78 | Present |
| D2 | Oribatida | *Eniochthonius minutissimus* | 229763 | 0,63 | Present |
| a11 | Oribatida | *Eueremaeus valkanovi* | 1401269 | 0,74 | Absent |
| P1_14 | Oribatida | *Eupelops occultus* | 2234141 | 0,77 | Absent |
| a13 | Oribatida | *Eupelops torulosus* | 198282 | 0,77 | Absent |
| P4_3 | Oribatida | *Euzetes globulus* | 334610 | 0,79 | Absent |
| P1_4 | Oribatida | *Fuscozetes fuscipes* | 1686651 | 0,77 | Present |
| P1_7 | Oribatida | *Galumna lanceata* | 229834 | 0,81 | Absent |
| D3 | Oribatida | *Galumna obvia* | 885392 | 0,79 | Absent |
| P1_8 | Oribatida | *Gustavia microcephala* | 1685391 | 0,65 | Absent |
| MI_402 | Oribatida | *Heminothrus targionii* | 2664691 | 0,77 | Present |
| a15 | Oribatida | *Hermanniella punctulata var. septentrionalis* | 885393 | 0,77 | Absent |
| a16 | Oribatida | *Liacarus coracinus* | 198285 | 0,7 | Present |
| a17 | Oribatida | *Liacarus xylariae* | 198284 | 0,81 | Present |
| MI_463 | Oribatida | *Licneremaeus licnophorus* | 999999011 | 0,77 | Absent |
| P1_16 | Oribatida | *Liebstadia similis* | 1250587 | 0,8 | Present |
| P4_5 | Oribatida | *Malaconothrus monodactylus* | 1797415 | 0,79 | Present |
| MI_464 | Oribatida | *Mesoplophora pulchra* | 334620 | 0,79 | Absent |
| MI_465 | Oribatida | *Metabelba pulverosa* | 229776 | 0,73 | Present |
| a18 | Oribatida | *Microtritia minima* | 229747 | 0,75 | Present |
| a19 | Oribatida | *Microzetes septentrionalis* | 999999012 | 0,74 | Absent |
| P1_10 | Oribatida | *Minunthozetes semirufus* | 1979919 | 0,77 | Present |
| P2_8 | Oribatida | *Nanhermannia comitalis* | 1979898 | 0,7 | Present |
| D4 | Oribatida | *Nanhermannia coronata cf.* | 198290 | 0,75 | Present |
| a20 | Oribatida | *Nanhermannia elegantula* | 66595 | 0,81 | Present |
| MI_408 | Oribatida | *Nanhermannia nana* | 198291 | 0,78 | Present |
| P1_11 | Oribatida | *Nothrus palustris* | 198293 | 0,74 | Present |
| MI_467 | Oribatida | *Nothrus silvestris* | 66602 | 0,78 | Present |
| P1_13 | Oribatida | *Oribatella quadricornuta* | 198298 | 0,75 | Absent |
| P1_18 | Oribatida | *Pantelozetes paolii* | 1979943 | 0,74 | Present |
| MI_412 | Oribatida | *Pergalumna nervosa* | 708370 | 0,78 | Absent |
| a88 | Oribatida | *Phthiracarus crinitus* | 229740 | 0,82 | Present |
| MI_492 | Oribatida | *Phthiracarus laevigatus* | 229740 | 0,81 | Present |
| a22 | Oribatida | *Porobelba spinosa* | 2886740 | 0,79 | Absent |
| a4_7 | Oribatida | *Punctoribates punctum* | 1720615 | 0,78 | Present |
| P4_9 | Oribatida | *Quadroppia maritalis* | 1250640 | 0,7 | Absent |
| a24 | Oribatida | *Scheloribates initialis* | 1979935 | 0,73 | Present |
| D8 | Oribatida | *Scheloribates latipes* | 1979937 | 0,72 | Absent |
| D7 | Oribatida | *Steganacarus magnus* | 52000 | 0,79 | Present |
| a4_11 | Oribatida | *Tectocepheus velatus* | 229869 | 0,69 | Present |
| P1_144 | Oribatida | *Zygoribatula exilis* | 1251916 | 0,76 | Present |
| a4_20 | Tardigrada | *Isohypsibius dastychi* | 947160 | 0,64 | Present |
| a3_3 | Tardigrada | *Paramacrobiotus richtersi* | 697321 | 0,57 | Present |

**Table S3.** Taxonomic assignments of animal GH45 cellulase orthologs found in NCBI RefSeq genomes.

| **Species name** | **NCBI Taxonomy ID** | **Accession number** | **Protein ID** | **NCBI Gene ID** | **Taxonomic assignment** |
| --- | --- | --- | --- | --- | --- |
| *Bradysia coprophila* | 38358 | GCF_014529535.1 | XP_037026636.1 | 119067642 | *Bradysia odoriphaga* |
|  |  |  | XP_037050424.1 | 119084512 | Protostomia |
|  |  |  | XP_037027558.1 | 119068175 | *Bradysia odoriphaga* |
| *Leptinotarsa decemlineata* | 7539 | GCF_000500325.1 | XP_023016322.1 | 111505702 | Chrysomelinae |
|  |  |  | XP_023029513.1 | 111517551 | *Gonioctena quinquepunctata* |
|  |  |  | XP_023016323.1 | 111505703 | Chrysomelinae |
|  |  |  | XP_023029514.1 | 111517551 | *Gonioctena quinquepunctata* |
|  |  |  | XP_023022929.1 | 111511149 | Chrysomelini |
|  |  |  | XP_023016326.1 | 111505705 | Chrysomelini |
| *Diabrotica virgifera virgifera* | 50390 | GCF_003013835.1 | XP_028147313.1 | 114340743 | Chrysomelidae |
|  |  |  | XP_028139473.1 | 114333726 | Chrysomelidae |
|  |  |  | XP_028143849.1 | 114337572 | Chrysomelidae |
|  |  |  | XP_028147314.1 | 114340743 | Chrysomelidae |
| *Anoplophora glabripennis* | 217634 | GCF_000390285.2 | XP_018561275.1 | 108903540 | *Anoplophora chinensis* |
|  |  |  | XP_018561265.1 | 108903530 | Lamiinae |
| *Dendroctonus ponderosae* | 77166 | GCF_000355655.1 | XP_019754618.1 | 109533680 | Dryophthorinae |
|  |  |  | XP_019754620.1 | 109533682 | Dryophthorinae |
|  |  |  | XP_019771468.1 | 109545306 | Cucujiformia |
|  |  |  | XP_019754619.1 | 109533681 | Dryophthorinae |
|  |  |  | XP_019766961.1 | 109542255 | Chrysomelinae |
| *Sitophilus oryzae* | 7048 | GCF_002938485.1 | XP_030751361.1 | 115878892 | Dryophthorinae |
|  |  |  | XP_030747083.1 | 115875708 | *Rhynchophorus ferrugineus* |
| *Thrips palmi* | 161013 | GCF_012932325.1 | XP_034236588.1 | 117642458 | *Frankliniella occidentalis* |
| *Frankliniella occidentalis* | 133901 | GCF_000697945.2 | XP_026287984.1 | 113213214 | *Thrips palmi* |
|  |  |  | XP_026287985.1 | 113213214 | *Thrips palmi* |
|  |  |  | XP_026289264.1 | 113214189 | *Thrips palmi* |
| *Folsomia candida* | 158441 | GCF_002217175.1 | XP_021945337.1 | 110843646 | Entomobryomorpha |
|  |  |  | XP_021948187.1 | 110845935 | Protostomia |

**Table S4. Training data for the fungal GH45 cellulase profile hidden Markov model.**

| **Species name** | **NCBI Taxonomy ID** | **Accession number RefSeq gene set** | **Protein IDs** |
| --- | --- | --- | --- |
| *Rhizoctonia solani* | 456999 | GCF_016906535.1 | XP_043186467.1 |
| *Pleurotus ostreatus* | 5322 | GCF_014466165.1 | XP_036634094.1 |
| *Marasmius oreades* | 181124 | GCF_018924745.1 | XP_043007043.1 |
| *Pseudozyma flocculosa PF-1* | 1277687 | GCF_000417875.1 | XP_007880076.1 |
| *Kalmanozyma brasiliensis GHG001* | 1365824 | GCF_000497045.1 | XP_016292070.1 |
| *Ustilago maydis 521* | 237631 | GCF_000328475.2 | XP_011388317.1 |

**Table S5**. RefSeq candidate cellulase orthologs lacking the Pfam Glyco_hydro_45 domain.

| **Spcies name** | **NCBI Taxonomy ID** | **Class** | **RefSeq accession number** | **Protein ID from inferred ortholog** |
| --- | --- | --- | --- | --- |
| *Osmia lignaria* | 473952 | Insecta | GCF_012274295.1 | XP_034173034.1 |
| *Bombus terrestris* | 30195 | Insecta | GCF_000214255.1 | XP_020720566.1  XP_012168823.1  XP_012168816.1  XP_012168808.1 |
| *Colletes gigas* | 935657 | Insecta | GCF_013123115.1 | XP_043266291.1  XP_043266289.1 |
| *Dufourea novaeangliae* | 178035 | Insecta | GCF_001272555.1 | XP_015435417.1 |
| *Rhagoletis zephyri* | 28612 | Insecta | GCF_001687245.1 | XP_017480646.1 |
| *Streptomyces lacrimifluminis* | 1500077 | Actinomycetes | GCF_014646095.1 | WP_189152206.1 |
| *Actinoplanes globisporus DSM 43857* | 1120949 | Actinomycetes | GCF_000379645.1 | WP_169516340.1 |
| *Brevibacterium jeotgali* | 1262550 | Actinomycetes | GCF_007828155.1 | WP_101587258.1 |
| *Aneurinibacillus danicus* | 267746 | Bacilli | GCF_007991215.1 | WP_146809708.1 |
| *Paenibacillus thalictri* | 2527873 | Bacilli | GCF_004307995.1 | WP_131011676.1 |
| *Chondromyces apiculatus DSM 436* | 1192034 | Deltaproteobacteria | GCF_000601485.1 | WP_197041519.1 |

**Table S6. Screened GenBank genomes.** Invertebrate genomes screened with fDOG or fDOG-Assembly for the presence of GH45 cellulases.

| **Species name** | **NCBI Taxonomy ID** | **Accession number** | **GH45 cellulase** | **Taxonomic group** |
| --- | --- | --- | --- | --- |
| *Sinella curviseta* | 187695 | GCA_004115045.3 | present | Collembola |
| *Tomocerus qinae* | 1765738 | GCA_020055645.1 | present | Collembola |
| *Yoshiicerus persimilis* | 1978554 | GCA_020352615.1 | present | Collembola |
| *Phaedon cochleariae* | 80249 | GCA_918026855.4 | present | Insecta |

Supplementary Files

File F1. GH45 cellulase gene tree in Newick format. For each species, only a single representative GH45 cellulase was selected. (uploaded separately)

File F2. GH45 cellulase gene tree of all detected GH45 cellulase co-orthologs Newick format (uploaded separately)
